# Suicidal thoughts and behaviors (STB) among psychiatric emergency patients at the emergency unit of a university hospital in Belgium (UZ Leuven) . A longitudinal approach with data from 2003-2015

**DOI:** 10.1192/j.eurpsy.2022.2189

**Published:** 2022-09-01

**Authors:** L. Van Eldere, S. Claes, W. Voorspoels, C. Yurdadon, M. Sabbe, R. Bruffaerts

**Affiliations:** 1 KU Leuven, Public Health Psychiatry, Leuven, Belgium; 2 UPC KU Leuven, Quality And Operational Policy, Kortenberg, Belgium; 3 KU Leuven, Mind- Body Research, Leuven, Belgium; 4 KU Leuven, Public Health And Primary Care, Leuven, Belgium

**Keywords:** Suicide, Suicidal thoughts and behaviors, Emergency Department

## Abstract

**Introduction:**

Suicidal thoughts and behaviors (STB) are a serious public health problem. Suicide prevention programs have been established over the years but many people who are suicidal do not seek treatment, and when they do they will end up in low-threshold sectors such as the Emergency Department in general hospitals. Previous studies about STB are mostly narrative, rather than a date-driven approach and limited in sample size.

**Objectives:**

The main goal of this study is to describe the prevalence and evolution of STB (ideation, plan or attempt) of the psychiatric patient referred to the Emergency Department of the University Hospital Gasthuisberg (Leuven, Belgium) over a 12 year period.

**Methods:**

During a 12 year period (2003-2015), all patients with a psychiatric referral to the Psychiatric Emergency Room (PER) of the University Hospital Gasthuisberg (Leuven, Belgium) were included (N˜25.000). We use descriptive statistics to summarize the data set, focusing on STB in terms of raw numbers, symptoms at referral, mental disorders and demographic characteristics.

**Results:**

Around 1/9 patients presents with suicide attempt; another 1/5 with suicidal thoughts. STB accounts for 35% of psychiatric primary complaints at the PER. Women were more likely to present with STB. The proportion of STB referrals remains stable over the years.

**Conclusions:**

Despite several reforms in mental health care, the PER remains a major entry point into mental healthcare for large proportions of STB patients.

**Disclosure:**

No significant relationships.

